# Alginate-Derivative Encapsulated Carbon Coated Manganese-Ferrite Nanodots for Multimodal Medical Imaging

**DOI:** 10.3390/pharmaceutics14122550

**Published:** 2022-11-22

**Authors:** Pemula Gowtham, Koyeli Girigoswami, Pragya Pallavi, Karthick Harini, Ilangovan Gurubharath, Agnishwar Girigoswami

**Affiliations:** 1 Medical Bionanotechnology, Faculty of Allied Health Sciences, Chettinad Hospital & Research Institute (CHRI), Chettinad Academy of Research and Education (CARE), Kelambakkam, Chennai 603103, India; 2 Department of Radiology, Chettinad Hospital & Research Institute (CHRI), Chettinad Academy of Research and Education (CARE), Kelambakkam, Chennai 603103, India

**Keywords:** manganese ferrite, carbon dots, alginate dialdehyde, oxidized sodium alginate, theranostics, MRI, multimodal imaging

## Abstract

Carbon-decorated ferrite nanodots (MNF@Cs) have been enhanced with superparamagnetism and higher fluorescence quantum yield by encapsulation with an alginate derivative to create a cost-effective and less toxic multimodal contrast agent for replacing the conventional heavy metal Gd-containing contrast agent used in MR imaging. The novel surface-engineered particles (MNF@C-OSAs), devoid of labels, can simultaneously provide both longitudinal and transverse relaxation-based magnetic resonance imaging (MRI) and fluorescence emission. According to the findings of in vitro studies, the calculated molar relaxivities and the molar radiant efficiencies are indicative of the multimodal efficacy of MNF@C-OSA as compared with MNF@C particles and conventional contrast agents used in medical imaging. MNF@C-OSAs were shown to be significantly biocompatible and negligibly toxic when assessed against A549 cells and zebrafish embryos, indicating their potential for use as theranostic agents.

## 1. Introduction

There are many different methods that are widely used to detect diseases in tissues, such as magnetic resonance imaging (MRI), fluorescence imaging, computed tomography (CT), photoacoustic imaging, positron emission tomography (PET), Raman imaging, and single photon emission CT (SPECT) [1,2,3,4]. MRI has become one of the most preferred methods in medicine today owing to its unique benefits in terms of noninvasiveness, penetration depths that are not limited, high resolution, and soft-tissue contrast as compared with other imaging technologies [5,6,7]. A structural MRI sequence of 1.5 T or more is usually used, depending on how deep the magnetic field is, to diagnose anatomical tumors and monitor the progress of therapeutic measures being taken in a clinical setting [8,9]. MRI sequences can be found in various formats, but T1-weighted and T2-weighted MRI are the most commonly used imaging sequences, anatomical structures being better displayed in T1-weighted images, while tumor types can be identified in T2-weighted images. It is often necessary to use MRI contrast agents in order to further enhance the sensitivity and resolution of scanned images [6,10,11]. A gadolinium (Gd)-based contrast agent can be used for T1 contrast to obtain more detailed boundary information for tumors, and 30 nm superparamagnetic iron oxide nanoparticles have been approved by the FDA as T2 contrast agents for prediction of the localization of therapeutic nanoparticles in tumor microenvironments based on MRI [12,13,14,15].

There are some safety concerns associated with gadolinium chelates, which are clinically proven and considered to be the best contrast agents for molecular MRI, due to their risk of causing nephrogenic systemic fibrosis in patients with renal dysfunction [16,17]. Iron oxide nanoparticles have seen considerable progress with regard to their design and use in a variety of biomedical applications. However, their inherent negative contrasting abilities restrict their use for MRI imaging [18,19]. Manganese chelates have also emerged as candidates for application as contrast agents in T1 imaging systems [20,21]. The use of manganese has some disadvantages, including the dechelation of manganese into free manganese ions, leading to the absence of properties associated with nanoparticles [22,23]. At the same time, the magnetic properties, catalytic capabilities, and dispersibilities of unprotected magnetic nanoparticles are diminished during catalytic transformations or in biomedical applications. There is a possibility that non-magnetic and inert chemically stable shells, such as silica or carbon shells, could effectively solve these problems by forming protective layers on magnetic nanoparticles [24,25]. As a promising approach, carbon-coated magnetic materials show better opportunities for ensuring excellent chemical and mechanical stabilities, whether these changes are caused by pH or temperature fluctuations in the cellular microenvironment [26,27]. Additionally, the outer carbon shell of manganese-based magnetic nanoparticles could be smoothly functionalized with various drugs and ligands, thereby providing efficient drug delivery and targeting of cancer cells [28,29,30,31].

The coating of carbon shells on the magnetic core stabilizes the magnetic properties along with the evolution of excellent optical properties that can help in the development of biosensors and fluorescence-based imaging agents [32,33]. By introducing oxygen defects on the surfaces of nanoparticles, carbon coating can increase absorption intensities in the visible light region. In addition to reducing uniaxial anisotropy on a particle’s surface, a carbon coating can also reduce the average blocking temperature. Making particles stable and dispersed is often difficult due to their poor surface chemical and physical properties [34]. As a result, surface engineering or functionalization of synthesized particles with various surface-stabilization agents, such as polymers, is necessary to overcome these shortcomings, allowing the particles to be stabilized, biodegradable, non-toxic, and have favorable surfaces for biocompatibility [25,35,36,37]. In particular, coatings with amphiphilic polymers were found to improve the stability, circulation half-life, efficacy of drug loading, and thereafter the fate of nanoparticles within the body, as well as their chemical properties. Polyethyleneimine, polyethylene glycol, chitosan, alginate, etc., are well-known polymers used commonly in the surface fabrication of nanoparticles [38,39]. Due to its excellent biodegradability, biocompatibility, and water solubility, sodium alginate has been approved by the FDA as a pharmaceutical adjuvant [40,41].

In the present study, carbon-coated manganese ferrite nanoparticles were synthesized by the hydrothermal procedure followed by entrapment in synthesized oxidized sodium alginate (OSA) hydrogels. OSA hydrogels have been chosen as suitable substitutes due to their ideal degradation rates and the biocompatibility features they possess. It is possible to take advantage of OSA in order to meet specific needs to deliver drugs, growth factors, and other payloads to diseased tissues. The fabricated particles can be considered potential candidates for multimodal MRI and optical imaging with negligible toxicity.

## 2. Materials and Methods

Manganese chloride tetrahydrate (MnCl_2_·4H_2_O), Ferric chloride hexahydrate (FeCl_3_·6H_2_O), Citric acid (C_6_H_8_O_7_), Urea (CH_4_N_2_O), and Doxorubicin hydrochloride were obtained from Sigma and used without any further purification. DMEM medium and antibiotic solutions, acridine orange, ethidium bromide, sodium alginate, sodium periodate, NaCl, calcium chloride, ethylene glycol, and ethanol were procured from HiMedia, Thane, India, and fetal bovine serum (FBS) was purchased from Gibco, Waltham, MA, USA. Double-distilled sterile water was used throughout the experimental procedure.

### 2.1. Synthesis of Carbon-Coated Ferrite Nanodots (MNF@Cs)

Carbon dots coated on ferrite were synthesized using a one-pot hydrothermal synthesis method that was reported previously [17]. The molar ratio of MnCl_2_·4H_2_O and FeCl_3_·6H_2_O was maintained at 1:2 by dissolving them in 50 mL of deionized water as molecular precursors for carbon dots; 3 g of citric acid and 1.5 g of urea were added to the initial solution. In a Teflon-coated stainless-steel autoclave, the mixture was heated to 160 °C for 12 h. To remove the unreacted components, the reaction mixture was centrifuged at 10,000 rpm for 15 min at room temperature. A hot-air oven at 160 °C was used to dry half of the resultant dispersion, which was then ground into powder for further characterization, while the other half was dispersed against water through a 1 kDa dialysis membrane overnight to purify it for further encapsulation.

### 2.2. Synthesis of Oxidized Sodium Alginate (OSA) and Encapsulation

Two grams of sodium alginate were dissolved in 120 mL of distilled water to produce OSA [42,43]. A 1:1 molar ratio of sodium periodate and sodium alginate was maintained by adding 20 mL of sodium periodate (NaIO_4_) to 60 mL of distilled water, followed by stirring for 24 h in the dark. A further 30 min of stirring in the darkroom was followed by the addition of 0.7 mL of ethylene glycol. A precipitate was obtained by adding 0.6 g NaCl and 200 mL ethanol to the solution. In order to obtain the desired solution, it was centrifuged at 8000 rpm for 20 min. Then, 100 mL of distilled water was used to dissolve the obtained precipitate of OSA. In order to obtain pure OSA in the form of white powder, 0.1 g of NaCl and 200 mL of acetone were added to the mixture, and the same wash step was repeated twice. It was then possible to air-dry the precipitate obtained and store it for further use. A quantity of 5 mL of synthesized MNF@C dots were added to the 16.75 mg/mL CaCl_2_ solution under continuous stirring, and 15 mg of OSA was dissolved in 5 mL of distilled water under continuous stirring. MNF@C solution was then added dropwise into the OSA solution for the completion of the encapsulation procedure, and stirring was maintained for 45 min. The encapsulated products were named MNF@C-OSAs. Doxorubicin was then added to the MNF@C-OSAs to maintain a final concentration of 1 mg/mL, followed by 30 min sonication for entrapment. The entrapment efficiency and release kinetics were measured spectrophotometrically [2].

### 2.3. Sample Characterization

A Shimadzu UV-1800 spectrophotometer was used to measure UV-Vis absorption spectra. Using the Malvern Nano Zs-90 size analyzer, we were able to investigate the size distribution and zeta potentials of the synthesized nanoparticles by applying the dynamic light scattering principle. An FTIR spectrometer from Bruker-Alpha was used to analyze synthesized nanoparticles for functional groups. The fluorescence spectrum was recorded with a Jasco FP-3800 spectrofluorometer. The time-resolved fluorescence was measured using the Fluoro Cube Lifetime System of HORIBA Jobin-Yvon, and the lifetime was calculated by fitting the spectra using the time-correlated single photon counting (TCSPC) method. A Bruker X-ray diffractometer X8 Kappa APEXII (Bruker, Bremen, Germany) was used to study crystal structures. A Lakeshore VSM 7410S was used to calculate magnetism magnitude. Additionally, GE Signa HDxT 1.5 T MRI was used to perform phantom and MR imaging, and an IVIS-Lumina LT, a PerkinElmer small-animal imaging system, was used for fluorescence imaging. Size analyses of the synthesized materials were carried out using a scanning electron microscope (SEM), FEI Quanta FEG200F, Hillsboro, OR, USA, and a Philips JEM-2000EX transmission electron microscope (TEM). We studied X-ray photoelectron spectra (XPS) with the VG Multi-lab 2000, which was manufactured by Thermo Scientific in the Waltham, MA, USA.

### 2.4. Toxicity Assessment

The toxicity of the prepared MNF@C-OSA particles was determined in comparison with uncoated MNF@Cs by treating A549 adenocarcinoma human alveolar basal epithelial cells. A549 cells were plated at 1 × 10^4^ cells per well in 24-well culture plates with Dulbecco’s Modified Eagle’s Medium (DMEM) along with 10% fetal bovine serum (FBS) and 1% antibiotic solution (HiMedia, India), maintaining a 5% CO_2_ level at 37 °C. The 3-(4,5-dimethylthiazol-2-yl)-2,5-diphenyltetrazolium bromide (MTT) assay was performed according to the standard protocol. For both attached and poorly attached cells, mitochondrial activity was measured using the MTT assay, which was proportional to cell viability. The MTT tetrazolium salt can be reduced only by biochemically active cells, whereas dead cells cannot perform this reduction reaction. The 10^4^ cells were seeded per well on poly-L-lysine coated plates and allowed to grow for 24 h, and different concentrations of both particles were added, followed by a further 24 h of incubation. To the cell-culture wells, the MTT salt solution in PBS was added at a concentration of 5 mg/mL after discarding the culture media. We incubated the MTT-treated cells at 37 °C for 4 h in a CO_2_ incubator. Optical density at 570 nm was measured after dissolving the formazan crystals with DMSO. As described by Metkar et al., the percentage of viability of cells was calculated [44]. A double-staining method with acridine orange (AO = 100 µg/mL) and ethidium bromide (EB = 100 µg/mL) was used to assay and take the fluorescence images of live and dead cells and identify apoptotic cells [45]. A549 cells were cultured in coverslips for 24 h supplemented with DMEM, 10% FBS, and 1% antibiotic solution at 37 °C in a humidified atmosphere. After 24 h, 4 μg/mL of MNF@C-OSA-Dox was added to the cells and further incubated for 24 h. The coverslips were taken on a grease-free glass sterile slide inside a biosafety cabinet, and a mixture of the two dyes (AO/EB) was added. The coverslips were incubated with the dye solution for 3 min at 37 °C in an incubator and visualized under a fluorescent microscope using appropriate filters. The green fluorescent cells represented live cells, and the red fluorescent cells represented dead cells.

A hemocompatibility test was performed using a hemolysis assay. After stabilizing fresh blood with EDTA, it was centrifuged for 15 min at 1500 rpm, the supernatant was removed, and the pellet was washed with PBS for 5 min to remove all serum particles. Red blood cells were then diluted ten times with PBS. A quantity of 0.1 mL of diluted RBC suspension was added to a 1.5 mL tube containing water (as a positive control), PBS (as a negative control), and MNF@C and MNF@C-OSA particles at various concentrations (2.5, 5, 10, 15, and 20 mM) in PBS. Samples were centrifuged for 1 min at 12,000 rpm after being incubated for two hours at 37 °C. We used the formula below to measure the absorbance at 541 nm and calculated the percentage of hemolysis in the supernatants.

$$\mathrm{Hemolysis}\;\left(\%\right)=\frac{A_{\left(sample\right)}-\;A_{\left(negative\;control\right)}}{A_{\left(positive\;control\right)}-\;A_{\left(negative\;control\right)}}\times 100\;$$

Further, toxicity was assessed in vivo using zebrafish embryos. In an aquarium, fish were kept at constant temperatures of 30 ± 1 °C at a light/dark ratio of 13/11 h [46]. Live brine shrimps were fed daily to the fish, along with dry flakes, and the pH of the tank was maintained at 7.4 ± 0.2. A breeding tank was set up with zebrafish males and females separated at a 2:1 ratio before mating. Eggs were collected from the mating tank the following day and rinsed with a sterile buffer two to three times. With the help of an inverted microscope, nearly 30 embryos in each of the treatment groups, along with the control, were observed as they developed in response to 5, 10, and 20 mM of MNF@C and MNF@C-OSA particles. The embryos were imaged and examined at 10, 24, 48, and 72 h post-fertilization (hpf) to monitor the developmental stages.

### 2.5. In Vitro MR and Optical Imaging

In 24-well plates, at room temperature, increasing concentrations of synthesized MNF@C and MNF@C-OSA particles were added to water phantoms. We imaged the phantoms using both T1- and T2-weighted MRI protocols on a 1.5 T GE Signa HDxT MRI scanner. In order to obtain slices of 2 mm thickness using T1-weighted FLAIR imaging, a TR of 3000 ms, a TE of 14 ms, an FOV of 24 × 24, and variable T1s between 400 and 2000 ms were used in the imaging sequence. We used a T2-weighted turbo spin-echo sequence to obtain slices of 2 mm thickness, which were conducted at variable TEs between 10 and 100 ms, where the TR was 100,000 ms, the FOV was 24 by 24, and the echo train length was 12 ms. In order to conduct phantom imaging, a fluorescence imaging system called IVIS was used. In a 96-well plate, samples were taken at varying concentrations for phantom imaging, and 460 nm-band optical filters were used to obtain fluorescent images.

## 3. Results and Discussion

**Figure 1:**
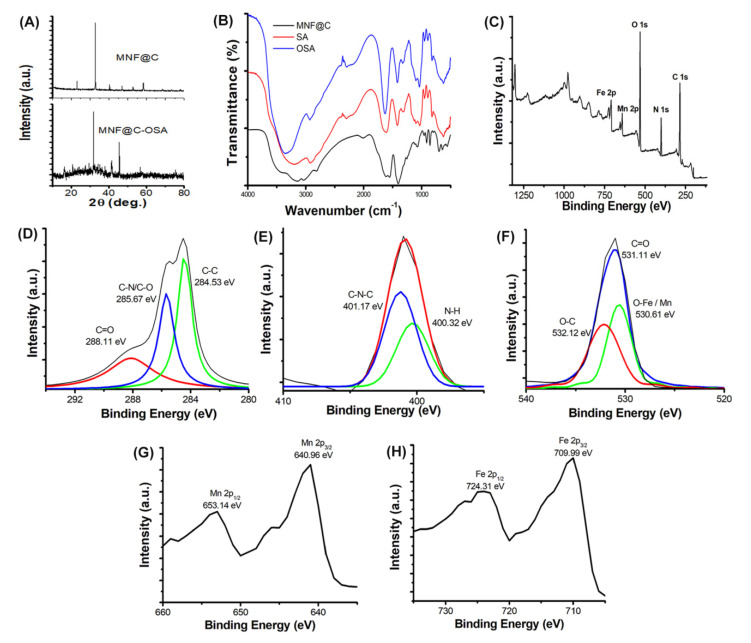
(**A**) XRD images of MNF@C and MNF@C-OSA dots. (**B**) FTIR spectra of MNF@Cs, sodium alginate (SA), and oxidized sodium alginate. (**C**) XPS image of MNF@Cs to confirm the structure and chemical composition. Fitted narrow-range XPS spectra of (**D**) C 1s, (**E**) N 1s, (**F**) O 1s, (**G**) Mn 2p, and (**H**) Fe 2p of MNF@Cs.

The crystalline structures of the MNF@C dots were examined by X-ray diffraction analysis (Figure 1A). The patterns showed a reflection that peaked at 22.26°, representing the turbostratic carbon phase of graphite containing a structural ordering of amorphous and crystalline graphite phases. However, the OSA-encapsulated MNF@C dots were amorphous, as indicated by the broad, noisy characteristics of their XRD peaks. A good agreement was also observed between the XRD peaks and manganese ferrite standard XRD patterns (JCPDS card no. 74-2403). As can be seen from the patterns, the peak that occurred at the highest point was the (311) plane of MnFe_2_O_4_ at 2θ = 33.69°. By applying the Scherrer equation, it was possible to calculate the lattice parameter and crystallite diameter of the MNF@Cs. The lattice parameter was 8.706 Ǻ, and the crystalline size obtained was 14.43 nm [47].

FTIR spectra were also acquired to probe the specific surface functional groups and chemical compositions of the MNF@C dots, SA, and OSA. It was confirmed that metal oxide was formed by the vibration of Fe-O bonds at 559 cm^−1^. Absorption bands at 3048 cm^−1^ and 3160 cm^−1^ corresponded to the vibrations of the aromatic C-H bonds stretching in the visible wavelength range (Figure 1B). A band at 1550 cm^−1^ and a band at 1616 cm^−1^ were the result of the N-H bending of amine groups. C-N stretching was observed at 1123 and 1252 cm^−1^. All the observed FTIR bands supported the decoration of the MNF@C dots. As a result of the stretching of hydroxyl groups, the alginate showed a characteristic band centered at approximately 3200 cm^−1^, and at 2931 cm^−1^ a low-intensity band was associated with -CH_2_ groups. The asymmetric and symmetric stretching modes of carboxylate groups were both seen at 1616 cm^−1^ and 1419 cm^−1^, respectively. Vibrations ranging between 1153 cm^−1^ and 1028 cm^−1^ corresponded to the C-O-C stretching of glycoside bonds in the polysaccharide. The spectral observations suggest that asymmetric and symmetric stretchings of carboxylate vibrations occurred at 1635 cm^−1^ and 1419 cm^−1^, respectively (Figure 1B). C=O stretching of aldehyde groups was signified by a feeble band at 1724 cm^−1^. The specific peak at 799 cm^−1^ indicated the -CH out of the plane. These characteristic bands suggest the synthesis of alginate dialdehyde or OSA.

XPS spectroscopy was used to further investigate structure and chemical composition (Figure 1C). At their respective binding energies, iron, manganese, carbon, oxygen, and nitrogen were observed in the XPS spectra of the full range. The photoelectron lines with binding energies of 724.31 and 709.99 eV were attributed to Fe 2p, while the photoelectron lines with binding energies of 653.14 and 640.96 eV were attributed to Mn 2p. It was confirmed that MnFe_2_O_4_ contained the metal oxides Fe-O and Mn-O by the characteristic peak at 530.61 eV. MNF@C dots may exhibit a peak at 532.12 eV as a result of metal-O-C bonds, and C=O may be responsible for the peak at 531.11 eV. The peaks at 401.17 eV and 400.32 eV represent C-N-C and N-H bonds, respectively. There were three important peaks (288.11 eV for C=O, 285.67 eV for C-N/C-O, and 284.53 eV for C-C) at the C 1s region of the XPS spectra, supporting the formation of the MNF@C dots (Figure 1D–H).

**Figure 2:**
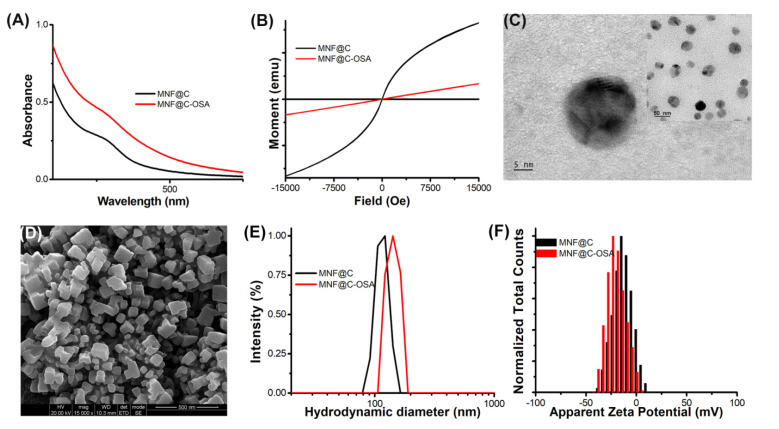
(**A**) Absorption spectra of MNF@Cs and MNF@C-OSAs. (**B**) Vibrating-sample magnetometry of MNF@Cs and MNF@C-OSAs. (**C**) Transmission electron microscope image of MNF@Cs; the inset shows a 50 nm scale. (**D**) Scanning electron microscope image of MNF@C-OSAs. (**E**). Colloidal particle size distribution of MNF@Cs and MNF@C-OSAs. (**F**) Measured zeta potentials of MNF@Cs and MN@C-OSAs.

As shown in Figure 2A, the synthesized MNF@C particles had a broad absorption peak in the range of approximately 430 nm to 470 nm, which was centered at 458 nm. The OSA-encapsulated MNF@C dots showed similar trends to the MNF@Cs in their absorption spectra, with higher intensities and being much outstretched. The change might have arisen due to the change in the microenvironment around the MNF@Cs, indicating encapsulation. The contrasting abilities of the MNF@C and MNF@C-OSA dots were validated via MR imaging by determining the magnetic properties of the synthesized MNF@C and MNF@C-OSA dots [48,49]. The lack of an apparent hysteresis loop in the VSM spectra indicated that the MNF@C and MNF@C-OSA dots were superparamagnetic, making them highly useful as contrast agents for MRI (Figure 2B).

TEM and SEM analyses confirmed the surface morphologies and crystal structures of the MNF@Cs and MNF@C-OSAs (Figure 2C,D). The transmission electron micrograph clearly shows the crystal structure of the MNF@Cs, the black spots in the center indicating the MNF core and the shadow for carbon shells. The SEM image of MNF@C-OSAs showed a uniform cubic structure due to the presence of polymers. The colloidal properties were evaluated by a particle size analyzer using the dynamic light scattering principle. The hydrodynamic diameter (dH) for MNF@Cs was found to be 113 nm, whereas the dH of MNF@C-OSAs was 140.7 nm (Figure 2E), with polydispersity indices of 0.23 and 0.39, respectively. The polydispersity index (PDI), a dimensionless parameter, was used to quantify the distribution of particle sizes. The PDI values were extrapolated from the autocorrelation function and ranged from 0.01 for monodispersed particles up to 0.7. The PDIs of the synthesized MNF@Cs and MNF@C-OSAs indicated monodispersity as per the above range. Zeta potentials were measured to study the surface charge-driven stabilities of the fabricated particles. The zeta potential of the MNF@Cs was −14.9 mV, whereas the zeta potential was −23.3 mV for MNF@C-OSAs (Figure 2F). The higher surface charge indicated the higher stability of the MNF@C-OSAs.

**Figure 3:**
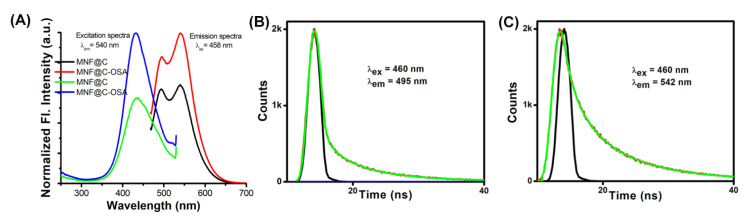
(**A**) Emission and excitation spectra of the MNF@C and MNF@C-OSA dots. (**B**) Time-resolved fluorescence image of MNF@C-OSAs at λ_em_ = 495 nm. (**C**) Time-resolved fluorescence image of MNF@C-OSAs at λ_em_ = 542 nm.

As shown in Figure 3A, the MNF@Cs exhibited steady-state fluorescence spectra upon excitation at 458 nm. There was an intriguing dual emissive behavior observed, with the particles peaking at 540 nm and a shoulder at 495 nm. Since the surfaces of the MNF@Cs were abundant with amine functional groups, fluorescence emissions were generated. As a result of urea being added to the reaction mixture, primarily to dope the nitrogen atoms, surface traps were passivated, which made the MNF@Cs fluorescent. Additionally, temperatures played critical roles, and below 200 °C were equally important for tuning the photoluminescence, because amino groups would abandon the surfaces at higher temperatures. Various emission centers or surface states of the carbon dots were thought to contribute to the dual emissive behavior of the MNF@Cs. There was a possibility that these surface states could be understood as so-called ‘traps’, and these traps were observed in the XPS spectrum as the result of different functional groups on the surface. The excitation spectra were recorded, keeping λ_em_ at 540 nm fixed. The obtained excitation spectra showed a clear peak at 433 nm. Similar steady-state fluorescence spectral behavior was observed for the MNF@C-OSAs with higher intensity. The increased intensity clearly supported the spectrophotometric observation of the entrapment of MNF@Cs in the polymeric matrix. The higher intensity gave an indication of a higher quantum yield that could be effective in optical-imaging-based diagnosis [50,51,52,53]. The absolute quantum yields for the MNF@Cs and MNF@C-OSAs were calculated, taking Rhodamine 6G in methanol as standard [54]. The values obtained were 0.52 ± 0.011 and 0.71 ± 0.014 for MNF@Cs and MNF@C-OSAs, respectively. The TCSPC method was used to analyze the fluorescence decay profiles of MNF@Cs, and triple exponential fitting was employed to fit the data (Figure 3B,C). This experiment was carried out with an excitation wavelength of 460 nm, and two emission wavelengths of 490 nm and 540 nm were used to measure the emissions. It was found that the particles emitted three different fluorescence decays, two (τ_1_ and τ_2_) of which had almost similar values for fluorescence decay time; however, the contribution values emitted by the particles differed from one another (Table 1). The τ_3_ value was 1.75 ns when emission was recorded at 495 nm, while the same was 5.39 ns at λ_em_ = 542 nm. In this study, the quantum confinement effect was largely responsible for the variation in contribution values.

### 3.1. Phantom MRI

**Figure 4:**
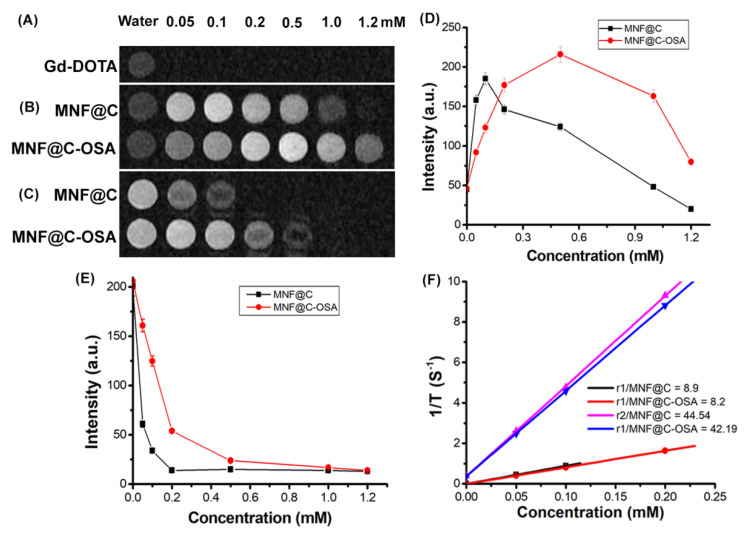
(**A**) T1-weighted phantom images of Gd-DOTA. (**B**) T1-weighted phantom images of MNF@C and MNF@C-OSA dots with varying concentrations; water was taken as a control. (**C**) T2-weighted phantom images of MNF@C and MNF@C-OSA dots with varying concentrations; water was taken as a control. (**D**) T1 image intensities for MNF-C and MNF@C-OSA dots were plotted against concentrations. (**E**) T2 image intensities for MNF-C and MNF@C-OSA dots were plotted against concentrations. (**F**) Relaxivities were plotted against concentrations to determine molar relaxivities.

By using phantom MR imaging, we evaluated the capabilities of the MNF@C and MNF@C-OSA dots to cause water proton relaxation changes. The MNF@C and MNF@C-OSA particles were taken in a 24-well plate with variable concentrations to monitor phantom MR imaging under a 1.5 T scanner. Water was taken as the control for this study. Figure 4 shows the T1- and T2-weighted phantom MR images of MNF@Cs and MNF@C-OSAs with increased concentrations. The intensities were measured using the DICOM image processing program. The MNF@Cs showed a positive contrasting effect due to the presence of Mn in the nanoparticles (Figure 4B). The intensities were increased to the concentration of 0.1 mM, and a further increase in concentration showed a sudden fall in intensity due to a quenching effect. A similar result was observed for the MNF@C-OSAs, but the limiting concentration differed from 0.1 mM for MNF@Cs to 0.5 mM for MNF@C-OSAs. The linear portions were used to calculate the molar relaxivities as 8.9 mM^−1^s^−1^ for MNF@Cs and 8.2 mM^−1^s^−1^ for MNF@C-OSAs. The quenching might have occurred because the MNF was packed into such small volumes that water molecules were unable to access the coordination sphere of Mn as a result. Furthermore, excess concentrations of MNF may have a detrimental effect on T1-weighted images due to a disproportionate weighting of the T2 effect. The MNF@C-OSAs showed a wide range due to the ability of polymers to hold the excess water molecules in the vicinity of the MNF. Therefore, a negative contrasting effect was observed with a higher concentration of MNF@C-OSAs, and the increment and quenching effects were smoother than for the MNF@Cs, making the MNF@C-OSAs better for use as T1 contrast agents in MRI. The phantom images of the commercially available MRI contrast agent Gd-DOTA were recorded in Figure 4A for comparison with the MNF@C-OSAs. The relaxivity of Gd-DOTA was 2.98 mM^−1^s^−1^, which was significantly less than those of the MNF@C and MNF@C-OSA particles.

The T2-weighted MR images are shown in Figure 4C. The MNF@Cs showed a negative contrasting effect due to the presence of Fe in their structures. The MNF@Cs showed a reduction in the intensities with increasing concentration and reached a trough at 0.2 mM, retaining the values with further increasing concentrations. A reduction in intensities or negative contrasting effects for the MNC@C-OSAs was observed up to a concentration of 1.0 mM. The MNF@Cs showed a steep decline in intensity at 0.05 mM, whereas the MNF@C-OSAs showed a gradual change in the entire range, which made them better T2 contrast agents than the unencapsulated particles, though the r2 value for MNF@Cs (44.54 mM^−1^s^−1^) was better than that for MNF@C-OSAs (42.19 mM^−1^s^−1^). The relaxivity ratios r2/r1 for MNF@Cs and MNF@C-OSAs were 5.01 and 5.14, which were in the limit of 10 > r2/r1 > 3, showing their potential for use as twin-mode T1 and T2 contrast agents.

### 3.2. Phantom Optical Imaging

**Figure 5:**
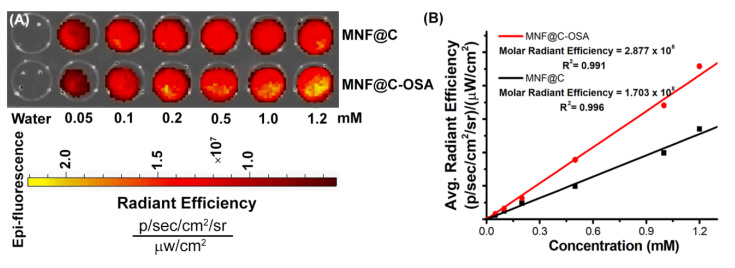
(**A**) IVIS-based fluorescent phantom images of MNF@Cs and MNF@C-OSAs at increasing concentrations. (**B**) The fluorescence image intensities were plotted against concentrations to calculate the molar radiant efficiencies.

Under the small-animal imaging system, the MNF@Cs and MNF@C-OSAs at various concentrations were imaged to demonstrate their optical capabilities as fluorescence imaging probes (Figure 5A). In this study, it was found that the fluorescence intensities of the MNF@C and MNF@C-OSA dots increased in accordance with their increasing concentrations, and all fluorescence image intensities were normalized to photons/second/centimeter^2^/steradian (p/s/cm^2^/sr), subtracting the corresponding background intensities. As MNF@C and MNF@C-OSA concentrations increased, the intensities increased linearly. According to Figure 5B, the average radiant efficiencies of the MNF@C and MNF@C-OSA dots with respect to concentrations are shown in the graph, where the slopes corresponding to the average radiant efficiencies were determined to be 1.703 × 10^8^ and 2.877 × 10^8^ p/s/cm^2^/sr per mM for the MNF@Cs and MNF@C-OSAs, respectively. The data clearly showed that the encapsulated particles had better radiant efficiencies compared to the unencapsulated particles. The obtained results were well in agreement with the steady-state fluorescence studies.

### 3.3. Toxicity Assessment

**Figure 6:**
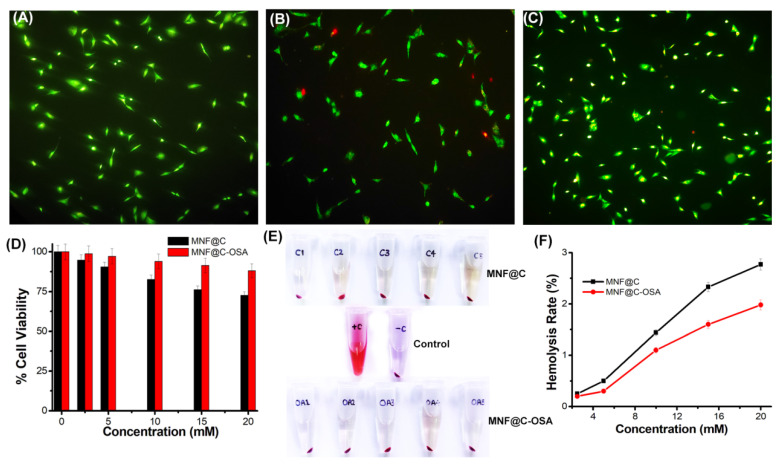
(**A**) Fluorescence microscopic image (10×) of A549 adenocarcinoma cells after staining with AO/EB. (**B**) AO/EB-stained A549 cells after treatment with 10 mM MNF@Cs. (**C**) AO/EB-stained A549 cells after treatment with 10 mM MNF@C-OSAs. (**D**) MTT assay using A549 cells to evaluate the toxicities of MNF@Cs and MNF@C-OSAs. (**E**) Hemocompatibilities of MNF@Cs and MNF@C-OSAs at varying concentrations (2.5, 5, 10, 15, and 20 mM). (**F**) Plot of percent hemolysis rates against varying concentrations of MNF@Cs and MNF@C-OSAs.

A double-staining procedure was used for a live/dead cell assay using AO/EB, in which live cells were stained green and dead cells were stained red. Figure 6A shows the control cells, where all cells were alive. Figure 6B,C show the 20 mM MNF@C- and MNF@C-OSA-treated cells, respectively. The MNF@C-treated cells showed 91 ± 2% cell viability after treatment with 10 mM MNF@Cs. In the case of the MNF@C-OSAs, the viability rate was better (97 ± 1%) compared to the MNF@Cs.

By performing an MTT assay on A549 adenocarcinoma human alveolar basal epithelial cells, we evaluated the in vitro cytotoxicities of the synthesized MNF@Cs and MNF@C-OSAs. According to the results of this study, the MNF@Cs were found to be more viable when coated with alginate derivatives than the uncoated MNF@Cs (Figure 6D). The OSA coating significantly reduced the toxic effects of the manganese present in the MNF@Cs, which might partially explain why the synthesized MNF@C-OSAs can be biocompatible and have negligible toxic effects. The hemocompatibility assessment results showed a similar situation, as reflected in the live/dead cell assay as well as in the MTT assay (Figure 6E). Figure 6 shows the superior hemocompatibility of MNF@C-OSAs with respect to MNF@Cs. Both types of particles showed hemolysis rates of less than 2% up to a 10 mM concentration. There was a slight increase in the hemolysis rate after 10 mM for MNF@Cs, whereas MNF@C-OSAs maintained a less than 2% hemolysis rate, even at a 20 mM concentration. Therefore, it can be concluded that both the particles are safe, but MNF-OSA showed enhanced safety for biomedical applications.

**Figure 7:**
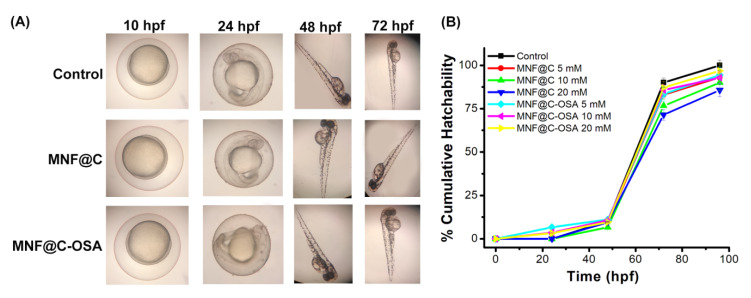
(**A**) Microscopic images of developmental stages of zebrafish embryos at the intervals of 10, 24, 48, and 72 hpf. (**B**) Percent cumulative hatchability of embryos was plotted against hpf, where untreated embryos were taken as controls.

Zebrafish embryos were used to validate the in vitro results. There were no abnormalities observed in the developmental stages of zebrafish embryos until 72 h, as shown in Figure 7A. The cumulative hatchabilities for the MNF@C-OSA-treated embryos showed almost similar results (95 ± 1.5%) to those for the controls at all three concentrations (Figure 7B). The percent hatchability was 91%, 90%, and 85.7% for the 5, 10, and 20 mM MNF@C-treated embryos, respectively (Figure 7B). The MNF@Cs caused minimal effects on embryo hatchability but did not cause developmental abnormalities in vivo.

**Figure 8:**
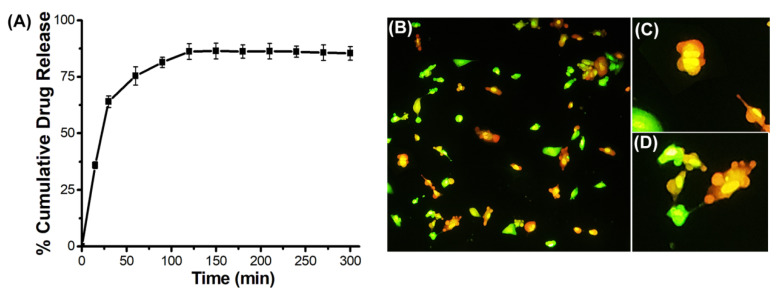
(**A**) Drug release profile for Dox-encapsulated MNF@C-OSAs. (**B**) AO/EB double-stained image of Dox-encapsulated MNF@C-OSA-treated A549 cells (10×). (**C**) Condensed nuclei (40×). (**D**) Membrane blebbing and release of apoptotic bodies formed from the treated cells (40×).

The anticancer drug doxorubicin (Dox) was encapsulated in MNF@C-OSA nanoparticles to evaluate the drug loading efficacy and therapeutic capabilities. The release kinetics were studied spectrophotometrically and plotted in Figure 8A. In the initial hour, there was a burst release of Dox, representing the removal of loosely bound Dox on the surface of polymer-encapsulated MNF@Cs. The release became a bit slower until 120 min compared to the initial hour, and later it followed the sustained release profile until the end of the study. A sustained release or zero-order kinetics profile is expected for any ideal drug delivery system. The entrapment efficiency was calculated spectrophotometrically, and it was 73%. The release was almost 87% after 300 min of the release profile study. The Dox-loaded particles were treated with A549 adenocarcinoma cells to evaluate therapeutic efficacy. Figure 8B shows the AO/EB double-staining fluorescence image that was taken after the incubation with Dox-entrapped MNF@C-OSAs. The image clearly shows that nearly 78% of cells were dead, and condensed nuclei and cell shrinkage, along with membrane blebbing with the release of apoptotic bodies, indicated apoptosis-driven cell death.

## 4. Conclusions

In this study, manganese ferrite-coated carbon dots were functionalized with an alginate derivative to improve biocompatibility with a reduction in toxicity. Several characterization techniques were applied in each stage of the development of the particles to confirm the structures, compositions, and physicochemical properties. The engineered particles have magnetic natures that were used in MRI to record twin T1- and T2-weighted images. The particles had fluorescent properties, too, due to the presence of carbon on their surfaces, and these were used to record fluorescence-based images. Due to their performances in multiple imaging modalities, the particles were found to be effective in multimodal diagnosis. The polymer OSA made the particles more biocompatible and bioavailable, which was reflected in the in vitro and in vivo studies using cell lines and zebrafish embryos, respectively. The assessment showed that the fabricated particles have negligible toxicities at higher concentrations compared to uncoated particles. The drug release profiles showed sustained release of the anticancer drug Dox for a longer period, which is an important parameter for an ideal drug delivery system. In conclusion, it can be stated from the above observations that the engineered MNF@C-OSA dots were found to be ideal for use as theranostic agents in multimodal diagnosis and cancer therapy.

## Figures and Tables

**Table 1:** Decay profile of MNF@C-OSAs.

λ_ex_/λ_em_ (nm)	τ_1_	a_1_	τ_2_	a_2_	τ_3_	a_3_	χ^2^
460/495	2.36	0.24	6.17	0.28	1.75	0.48	0.98
460/542	2.58	0.38	6.53	0.48	5.39	0.14	1.01

## Data Availability

All data are available in the manuscript.
